# 基于自建数据库的超高效液相色谱-四极杆-飞行时间质谱法测定谷物中7种真菌毒素

**DOI:** 10.3724/SP.J.1123.2023.07014

**Published:** 2023-11-08

**Authors:** Luxing ZHANG, Zheng ZHOU, Lin CAO, Jiang QIAN

**Affiliations:** 1.浙江药科职业大学, 浙江 宁波 315100; 1. Zhejiang Pharmaceutical University, Ningbo 315100, China; 2.宁波市药品检验所, 浙江 宁波 315048; 2. Ningbo Institute for Drug Control, Ningbo 315048, China

**Keywords:** 超高效液相色谱-四极杆-飞行时间质谱, QuEChERS, 真菌毒素, 稻谷, 小麦, ultra performance liquid chromatography-quadrupole-time of flight mass spectrometry (UPLC-Q-TOF/MS), QuEChERS, mycotoxins, rice, wheat

## Abstract

建立了基于自建数据库的超高效液相色谱-四极杆-飞行时间质谱(UPLC-Q-TOF/MS)测定水稻、小麦中7种真菌毒素的方法。样品经0.2%甲酸水-乙腈(50∶50, v/v)提取,用QuEChERS盐包脱水盐析,用HSS T3色谱柱(100 mm×2.1 mm, 1.8 μm)分离。采用UPLC-Q-TOF/MS在全信息串联质谱(MS^E^)模式下对样品进行筛查,针对筛查结果为阳性的样品,采用ESI源,在正、负离子飞行时间多反应监测(TOF-MRM)模式下进一步检测,用基质匹配标准曲线进行校准定量。结果表明,7种真菌毒素在各自范围内具有良好的线性关系,相关系数(*r*)为0.9900~0.9998。7种真菌毒素的检出限为0.50~400 μg/kg,定量限为1.00~800 μg/kg。水稻和小麦基质在低、中、高3个加标水平下的平均回收率分别为88.1%~123.9%和102.0%~123.4%; RSD分别为0.2%~13.6%和0.8%~14.8%。结果表明,在46批水稻中筛查出了黄曲霉素B_1_(AFB_1_)和黄曲霉素B_2_(AFB_2_),筛出率各为2.2%,其中有1批样品AFB_1_超过限量;在24批小麦中筛查出了脱氧雪腐镰刀菌烯醇(DON)和玉米赤霉烯酮(ZEN),筛出率分别为37.5%和 79.2%,均未超过限量。本方法采用MS^E^进行定性筛查,避免干扰物因质量数和保留时间都与目标物接近形成的假阳性现象,对筛查阳性的真菌毒素采用TOF-MRM模式进行定量。该法具有快捷、准确、灵敏度高的特点,适用于水稻、小麦样品中真菌毒素残留的分离和定量检测,可为水稻、小麦中真菌毒素污染监测和风险预警提供有力的技术支持。

## Abstract (en)

A method for the determination of seven mycotoxins in rice and wheat by ultra performance liquid chromatography-quadrupole-time of flight mass spectrometry (UPLC-Q-TOF/MS) based on self-built database was established. Samples were extracted with 0.2% formic acid aqueous solution-acetonitrile (50∶50, v/v), dehydrated and salted according to the QuEChERS method (4 g of magnesium sulfate, 1 g of sodium chloride, 1 g of sodium citrate, 0.5 g of citrate disodium salt), and separated on an HSS T3 column (100 mm×2.1 mm, 1.8 μm). UPLC-Q-TOF/MS with MS^E^ screening was performed, and the positive and negative ions of the screened mycotoxins were calibrated and quantified using matrix-matched standard curves with time of flight multiple reaction monitoring (TOF-MRM). The results showed that aflatoxin G_1_ (AFG_1_), aflatoxin G_2_ (AFG_2_), aflatoxin B_1_ (AFB_1_), aflatoxin B_2_ (AFB_2_), and ochratoxin A (OTA) exhibited moderate matrix effects in rice, while OTA and zearalenone (ZEN) exhibited moderate matrix effects in wheat. The seven mycotoxins showed good linearities in their respective concentration ranges, with correlation coefficients (*r*) of 0.9900-0.9998. The limits of detection (LODs) for rice and wheat were 0.50-400 and 0.50-200 μg/kg, respectively, and the limits of quantification (LOQs) for both cereals were 1.00-800 μg/kg. In rice, the average recoveries at three spiked levels of low, medium, and high were 88.1%-123.9%, with relative standard deviations (RSDs) of 0.2%-13.6%. In wheat, the average recoveries at three spiked levels of low, medium, and high were 102.0%-123.4%, with RSDs of 0.8%-14.8%. As a result, one batch of 46 batches of rice was screened out for AFB_1_ and AFB_2_, with a screening rate of 2.2%, of which the measured values were 10.8 μg/kg and 1.2 μg/kg, respectively. According to GB 2761-2017, the maximum allowable level of AFB_1_ in rice is 10 μg/kg; thus, the exceeding rate for AFB_1_ is 2.2%. Deoxynivalenol (DON) was screened out in 9 out of 24 batches of wheat (screening rate, 37.5%), while ZEN was screened out in 19 batches (screening rate, 79.2%). According to GB 2761-2017, the maximum allowable levels of DON and ZEN in wheat are 1000 and 60 μg/kg, respectively. The levels of DON and ZEN detected in the wheat samples did not exceed these limits. The proposed method uses MS^E^ for qualitative screening to avoid the occurrence of false positives caused by interfering compounds with mass numbers and retention times similar to those of the analytes. TOF-MRM mode is then used to quantify the positively screened mycotoxins. The method is fast, accurate, sensitive, and suitable for the isolation and quantitative detection of mycotoxin residues in rice and wheat samples. The findings provide powerful technical support for mycotoxin contamination monitoring in rice and wheat and early risk-warning efforts.

真菌毒素造成的污染是食品安全较为重要的问题之一,植物在种植、生长、收获、加工、运输等各个环节均有被污染的可能,根据联合国粮食及农业组织的统计,全球25%的食品受到不同程度的真菌毒素污染,这已经成为一个全球性的问题^[1⇓-3]^。目前发现的数百种真菌毒素中,约有12种真菌毒素被认为具有严重健康危害,其中黄曲霉素B_1_(AFB_1_)被列为Ⅰ类致癌物^[4]^。

前期,本课题组应用超高效液相色谱-四极杆-飞行时间质谱(UPLC-Q-TOF/MS)建立了水稻、小麦中18种真菌毒素的高分辨质谱数据库,可以在无对照品的状态下,准确筛查出水稻、小麦中痕量的真菌毒素,建立了良好的非靶向定性方法^[5]^。参考GB 2761-2017《食品安全国家标准 食品中真菌毒素限量》^[6]^和EC No. 1881/2006^[7]^,在稻谷、小麦基质中有明确监管限量要求的真菌毒素有7种,对这7种真菌毒素有必要进行定量分析,确定是否符合标准限度要求,保护公众食品安全。

目前定量首选的方法是液相色谱-三重四极杆质谱法(LC-MS/MS)^[8,9]^,其具有较高的灵敏度和选择性^[10,11]^,但为了准确定性和定量真菌毒素,需要获得高浓度对照品和相应的同位素内标^[12]^,有些对照品如AFB_1_纯度高、毒性大,不可避免会对实验者及实验环境造成一定危害,而且靠预先设定的对照品来进行定性,无法获取对照品以外的真菌毒素残留信息^[13]^。另外,LC-MS/MS分辨力有限,容易产生假阳性现象。高分辨质谱(HRMS)可在全扫描模式下采集选定质量范围内的全部离子,提供大量的定性信息。随着技术的发展,HRMS选择性、动态范围、线性、灵敏度等有了很大的提升,从而扩大了在定量分析中的应用^[14,15]^。四极杆-飞行时间质谱兼具四极杆的高选择性和飞行时间质谱的高分辨率,数据采集可以兼容定量分析的灵敏度和选择性。

本文基于UPLC-Q-TOF/MS,建立了水稻、小麦基质中7种真菌毒素的测定方法。采用UPLC-Q-TOF/MS在全信息串联质谱(MS^E^)模式下对样品进行筛查,针对筛查结果为阳性的样品,采用ESI源,在正、负离子飞行时间多反应监测(TOF-MRM)模式下进一步检测,用基质匹配标准曲线进行校准定量,为水稻、小麦中真菌毒素污染监测和风险预警提供有力的技术支持。

## 1 实验部分

### 1.1 仪器与试药

G2 XS四极杆-飞行时间高分辨质谱仪、ACQUITY UPLC超高效液相色谱仪、MassLynx V4.2工作站、UNIFI科学信息学系统(美国Waters公司); Milli-Q超纯水纯化系统(美国Millipore公司);电子天平(瑞士METTLER TOLEDO公司); L580离心机(上海卢湘仪离心机仪器有限公司); LPD2500振荡器(莱普特科学仪器(北京)有限公司)。

黄曲霉素B_1_(10.0 μg/mL)、黄曲霉素B_2_(AFB_2_, 10.0 μg/mL)、黄曲霉素G_1_(AFG_1_, 10.0 μg/mL)、黄曲霉素G_2_(AFG_2_, 10.0 μg/mL)、赭曲霉素(OTA, 100 μg/mL)、脱氧雪腐镰刀菌烯醇(DON, 1000 μg/mL)标准溶液,玉米赤霉烯酮(ZEN, 10.0 mg)标准品均购自阿尔塔科技有限公司;QuEChERS萃取盐包购自美国安捷伦科技公司;甲醇、乙腈(质谱纯)购自德国Merck公司;乙酸、甲酸、乙酸铵(质谱纯)均购自阿拉丁试剂;实验用水为Milli-Q超纯水。

### 1.2 色谱-质谱分析条件

#### 1.2.1 UPLC条件

色谱柱为Waters HSS T3柱(100 mm×2.1 mm, 1.8 μm),柱温40 ℃,流动相A为含5 mmol/L乙酸铵的1%乙酸水溶液,流动相B为甲醇,流速为0.3 mL/min,样品进样量为10 μL。梯度洗脱程序见表1。

**表1:** UPLC梯度洗脱程序

t/min	φ(A)/%	φ(B)/%
0	90	10
2.0	90	10
3.0	80	20
7.0	76	24
10.5	70	30
13.5	40	60
15.0	30	70
18.0	25	75
18.1	5	95
21.9	5	95
22.0	90	10

A: 1% acetic acid aqueous solution containing 5 mmol/L ammonium acetate; B: methanol.

#### 1.2.2 Q-TOF/MS条件

ESI正离子模式:毛细管电压3.00 kV,锥孔电压40 V,离子源温度110 ℃,脱溶剂气温度450 ℃,锥孔气流速50 L/h,脱溶剂气流速800 L/h。ESI负离子模式:毛细管电压2.50 kV,锥孔电压40 V,离子源温度110 ℃,脱溶剂气温度450 ℃,锥孔气流速50 L/h,脱溶剂气流速800 L/h。LockSpary溶液:亮氨酸脑啡肽(正离子*m/z* 556.2771,负离子*m/z* 554.2615);采集模式:MS^E^、TOF-MRM模式。MS^E^模式设置:质量扫描范围*m/z* 50~1200;扫描时间0.25 s;低碰撞能量(LE)关闭,高碰撞能量(HE)为3~40 eV; TOF-MRM模式设置:详见表2中的参数设置。数据采集由MassLynx V4.2工作站完成。

**表2:** 7种真菌毒素的质谱参数

No.	Compound	Transitions (m/z)	Cone voltage/V	Collision energy/eV	Ion mode
1	aflatoxin B_1_ (AFB_1_)	329.068/311.053^*^; 329.068/243.066	40	20	[M+H]^+^
2	aflatoxin B_2_ (AFB_2_)	331.080/313.067^*^; 331.080/285.070	40	25	[M+H]^+^
3	aflatoxin G_1_ (AFG_1_)	313.070/285.072^*^; 313.070/270.050	40	25	[M+H]^+^
4	aflatoxin G_2_ (AFG_2_)	315.085/287.090^*^; 315.085/259.055	40	25	[M+H]^+^
5	ochratoxin A (OTA)	404.088/358.078; 404.088/239.005^*^	40	25	[M+H]^+^
6	deoxynivalenol (DON)	355.141/295.116; 355.141/265.103^*^	40	10	[M+CH_3_COO]^-^
7	zearalenone (ZEN)	317.138/273.146; 317.138/175.036^*^	40	25	[M-H]^-^

* Quantitative ions.

### 1.3 样品前处理

准确称取5.0 g样品,于50 mL聚丙烯具塞离心管中,加入20 mL 0.2%甲酸水-乙腈(50∶50, v/v),涡旋混匀,振荡提取25 min,加入QuEChERS萃取盐包(含4 g硫酸镁、1 g氯化钠、1 g柠檬酸钠、0.5 g柠檬酸二钠盐),振荡5 min,以4000 r/min的转速离心5 min,取上清液,用0.22 μm聚四氟乙烯滤膜过滤,取续滤液,进样分析。

### 1.4 标准溶液配制

分别精密量取AFG_1_、AFG_2_、AFB_1_、AFB_2_、OTA、DON标准品原溶液1 mL,置于5 mL容量瓶中,用乙腈定容至刻度;ZEN标准品用乙腈定量溶解至10 mL容量瓶中,配制7种真菌毒素标准储备液,于-20 ℃避光保存。取7种真菌毒素标准储备液适量,用乙腈稀释制成AFG_1_、AFG_2_、AFB_1_、AFB_2_、OTA、DON、ZEN质量浓度分别为50.0、50.0、50.0、50.0、200、40000、2000 ng/mL的混合标准溶液,于-20 ℃避光保存。

按课题组前期^[5]^确定的筛查方法,选用未检出真菌毒素的样品作为空白样品,按1.3节处理得到空白基质溶液,取7种真菌毒素混合标准溶液适量,用空白基质溶液稀释,配制成基质匹配混合校准溶液,其中AFG_1_、AFG_2_、AFB_1_、AFB_2_的质量浓度分别为0.25、0.50、0.75、1.00、1.25 ng/mL, OTA为1.00、2.00、3.00、4.00、5.00 ng/mL, DON为200、400、600、800、1000 ng/mL, ZEN为10.0、20.0、30.0、40.0、50.0 ng/mL。

### 1.5 UNIFI筛查条件

参照本课题组前期^[5]^研究,筛查条件为:选取加合物(包括[M+H]^+^、[M+NH_4_]^+^、[M-H]^-^和[M+CH_3_COO]^-^)作为目标质量,低能量通道质谱报告强度阈值100,高能量通道质谱报告强度阈值20;绝对保留时间漂移0.3 min;离子测得质量数与数据库中的离子理论质量数匹配容差5×10^-6^。

## 2 结果与讨论

### 2.1 色谱条件的优化

#### 2.1.1 色谱柱的选择

实验比较了BEH HILIC C18(100 mm×2.1 mm, 1.7 μm)和HSS T3(100 mm×2.1 mm, 1.8 μm)色谱柱,使用BEH HILIC C18色谱柱时,化合物出峰速度过快,其中DON几乎无保留,使用后者,化合物峰形和保留时间较为合理。推测HSS T3柱具有较高的硅烷亲和性,能增强极性化合物的保留。因此选择HSS T3色谱柱(100 mm×2.1 mm, 1.8 μm)。

#### 2.1.2 流动相条件的优化

本实验比较了水-甲醇体系、1%甲酸水溶液-甲醇体系、1%乙酸水溶液-甲醇体系与LS/T 6133-2018《粮油检验主要谷物中16种真菌毒素的测定 液相色谱-串联质谱法》标准中的含5 mmol/L乙酸铵的1%乙酸水溶液-甲醇体系。结果显示,采用含5 mmol/L乙酸铵的1%乙酸水溶液-甲醇体系时,得到的离子响应强度和峰形最优,提取离子色谱图如图1所示。

**图1:**
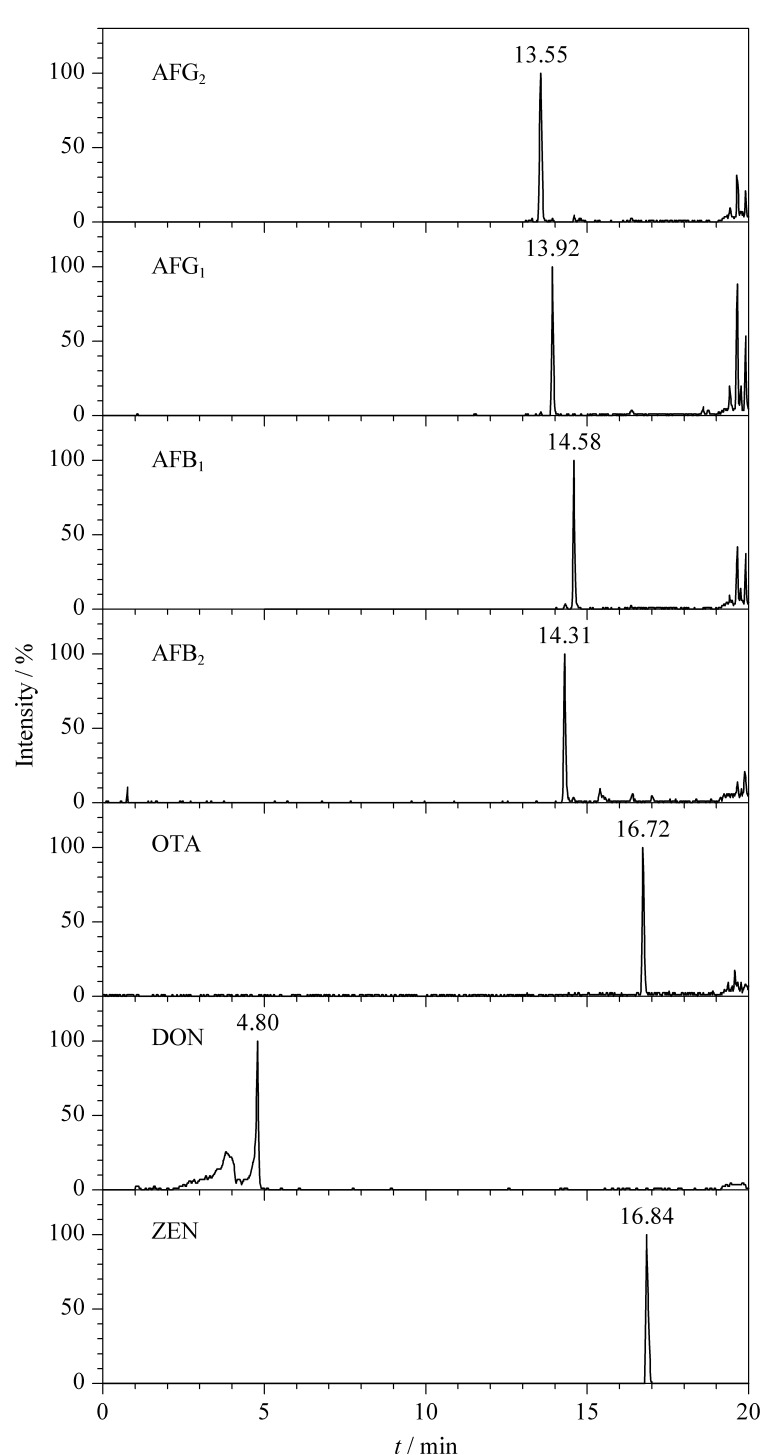
7种真菌毒素混合标准溶液的提取离子色谱图

### 2.2 质谱条件优化

7种真菌毒素中AFG_1_、AFG_2_、AFB_1_、AFB_2_、OTA采用正离子模式,DON、ZEN采用负离子模式,若均以正离子或负离子模式电离,会导致信号响应值达不到最佳。

本实验对MS^E^和TOF-MRM 2种数据采集模式进行比较,在色谱条件、离子源参数和积分方法一致的前提下,采用TOF-MRM模式获得的7种真菌毒素信号强度高、基线噪声小,有更好的灵敏度和选择性,适用于定量分析。MS^E^模式能够获得质量数精准的完整碎片信息,适于定性筛查分析。以AFB_1_为例,TOF-MRM模式和MS^E^模式所获得的质谱图对比如图2所示,该结果清楚展示了两种采集模式的差异。

**图2:**
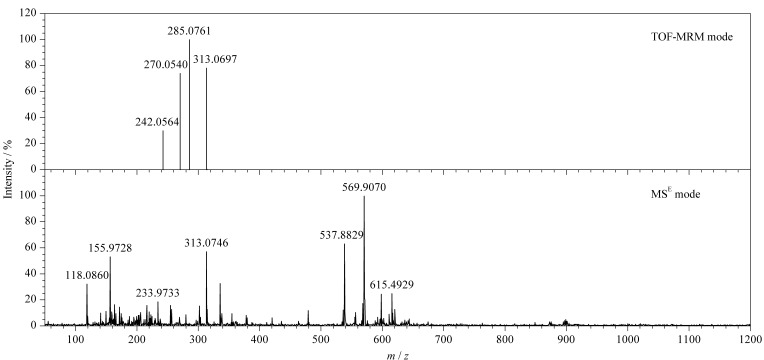
AFB_1_在TOF-MRM与MS^E^模式下的质谱图

### 2.3 前处理方法的优化

#### 2.3.1 提取溶剂酸度的选择

向水稻、小麦空白基质中定量加入7种真菌毒素标准品,考察提取溶剂甲酸水溶液-乙腈(50∶50, v/v)中甲酸的体积分数(0、0.2%、0.4%、0.8%、2.0%)对回收率的影响。结果如图3所示,与不添加甲酸相比,加入甲酸后目标化合物的回收率整体有所提高,甲酸体积分数为0.2%和0.4%时,回收率较高且接近。随着甲酸体积分数的增加,正离子模式下黄曲霉素类、OTA回收率逐渐降低,而负离子模式下DON和ZEN回收率基本不变,综合考虑,最终选择甲酸体积分数为0.2%。

**图3:**
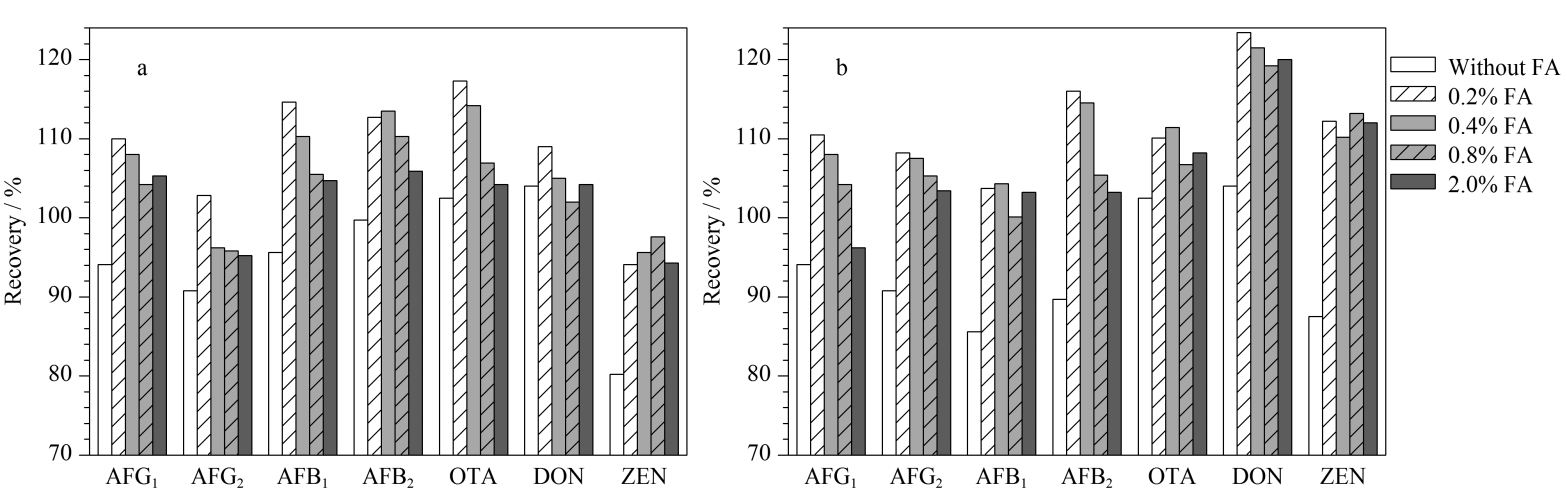
提取溶剂中甲酸的体积分数对(a)水稻和(b)小麦基质中真菌毒素回收率的影响

#### 2.3.2 净化方式的选择

以水稻、小麦空白基质加标样品的回收率为考察对象,以0.2%甲酸水溶液-乙腈(50∶50, v/v)为稀释液,比较了直接提取稀释法、MycoSpin 400净化柱法、QuEChERS萃取盐包结合Captiva EMR-Lipid过滤柱、QuEChERS萃取盐包结合Oasis PRIME HLB小柱和QuEChERS萃取盐包直接净化法。结果表明,用直接提取稀释法提取液离心后取上清液,过0.22 μm滤膜,7种真菌毒素的绝对响应较低,而且随着进样次数的增加目标物信号响应逐渐降低直到淹没在基线噪声中,因此后续不再考察。

对于其他净化法的考察结果如图4所示。QuEChERS萃取盐包直接净化法中AFG_1_、AFG_2_、AFB_1_、AFB_2_、ZEN的回收率比采用QuEChERS萃取盐包结合净化柱法(Captiva EMR-Lipid过滤柱、Oasis PRIME HLB小柱)更高。可能的原因是填料EMR和HLB本身对脂质有较强的去除效果,对ZEN中的大环类结构有较强的吸附作用,对AFG_1_、AFG_2_、AFB_1_、AFB_2_中的双呋喃环香豆素结构可能也有一定的吸附作用。通过几种前处理方法的比较,我们选用QuEChERS萃取盐包直接净化法,操作简便,回收率高。

**图4:**
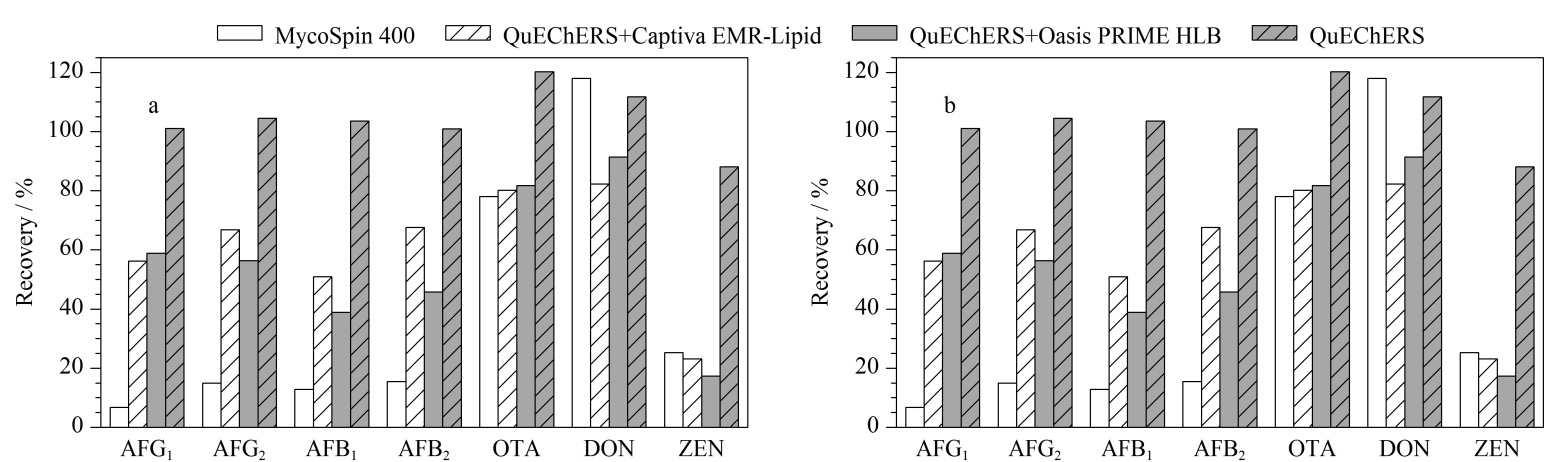
净化方法对(a)水稻和(b)小麦基质中真菌毒素回收率的影响

### 2.4 基质效应

分别用提取溶剂和基质溶液配制纯溶剂标准溶液和基质匹配混合校准溶液,以目标组分色谱峰面积对质量浓度绘制标准曲线。ME=(基质匹配校准曲线的斜率-溶剂标准曲线的斜率)/溶剂标准曲线的斜率×100%,|ME|≤20%为弱基质效应,可以忽略;20%<|ME|≤50%为中等基质效应;|ME|>50%为强基质效应。结果表明,在水稻基质中AFG_1_、AFG_2_、AFB_1_、AFB_2_和OTA具有中等强度的基质效应;在小麦基质中仅有OTA和ZEN为中等强度的基质效应。因此需采用基质匹配校准曲线进行校准定量。

### 2.5 标准曲线、检出限和定量限

前期,课题组已在UNIFI上建立了谷物中的18种真菌毒素数据库,并在无标准品的情况下,准确筛查出谷物中的真菌毒素残留,建立了良好的非靶向定性方法^[5]^。当筛查出的真菌毒素明显高于或低于限量时,容易得出准确的评判结论,但当其响应接近限量时,定量测定的准确性对结果的判定尤为重要,因此建立了包含限量浓度的标准曲线。如表3所示,本文根据GB 2761-2017和EC No. 1881/2006标准对谷物类及其制品的要求,合计对7种真菌毒素规定了限量,选取两个标准中较低的最大残留限量(MRL)作为本试验的限量标准。取1.4节的基质匹配混合校准溶液,按照1.2.1和1.2.2节方法进行测定,以目标组分色谱峰面积对质量浓度绘制标准曲线。

**表 3:** 7种真菌毒素的最大限量

Compound	GB 2761-2017			EC No.1881/2006			This work	
	Rice	Wheat		Rice	Wheat		Rice	Wheat
AFG_1_	none	none		total: 4.0	total: 4.0		total: 4.0	total: 4.0
AFG_2_	none	none						
AFB_1_	10	5.0						
AFB_2_	none	none						
OTA	5.0	5.0		5.0	5.0		5.0	5.0
DON	none	1000		1250	1750		1000	1000
ZEN	none	60		100	100		60	60

The total maximum limit for AFG_1_, AFG_2_, AFB_1_ and AFB_2_ was 4.0 μg/kg.

结果如表4、表5所示,在水稻和小麦基质中,各化合物在其线性范围内呈现良好的线性关系,相关系数(*r*)≥0.9900,以定量离子信噪比大于3计算检出限,为0.50~400 μg/kg,以定量离子信噪比大于10计算定量限,为1.00~800 μg/kg。

**表 4:** 水稻基质中7种真菌毒素的线性范围、线性方程、相关系数、检出限、定量限和基质效应

Compound	Linear range/(μg/L)	Linear equation	r	LOD/(μg/kg)	LOQ/(μg/kg)	|ME|/%
AFG_1_	0.25-1.25	y=2.12×10^3^x-3.69×10^2^	0.9945	0.50	1.00	46.2
AFG_2_	0.25-1.25	y=1.69×10^3^x-1.48×10^2^	0.9950	0.50	1.00	37.4
AFB_1_	0.25-1.25	y=1.74×10^3^x-0.87×10^2^	0.9953	0.50	1.00	48.1
AFB_2_	0.25-1.25	y=1.76×10^3^x+0.46×10^2^	0.9900	0.50	1.00	45.3
OTA	1.00-5.00	y=1.80×10^2^x-1.11×10^2^	0.9900	2.00	4.00	21.5
DON	200-1000	y=6.04x-6.04×10^2^	0.9934	400	800	13.4
ZEN	10.0-50.0	y=1.12×10^3^x-1.48×10^3^	0.9974	2.00	8.00	0.7

y: peak area; x: mass concentration, μg/L.

**表5:** 小麦基质中7种真菌毒素的线性范围、线性方程、相关系数、检出限、定量限和基质效应

Compound	Linear range/(μg/L)	Linear equation	r	LOD/(μg/kg)	LOQ/(μg/kg)	|ME|/%
AFG_1_	0.25-1.25	y=3.70×10^3^x-2.42×10^2^	0.9974	0.50	1.00	6.3
AFG_2_	0.25-1.25	y=2.23×10^3^x+1.52×10^2^	0.9934	0.50	1.00	17.3
AFB_1_	0.25-1.25	y=3.06×10^3^x-1.55×10^2^	0.9963	0.50	1.00	8.6
AFB_2_	0.25-1.25	y=3.52×10^3^x-3.17×10^2^	0.9972	0.50	1.00	10.5
OTA	1.00-5.00	y=1.72x+1.74×10^2^	0.9867	2.00	4.00	25.2
DON	200-1000	y=5.88x-3.08×10^2^	0.9961	200	800	15.6
ZEN	10.0-50.0	y=1.56×10^3^x+8.08×10^2^	0.9998	0.50	1.00	28.0

### 2.6 回收率和精密度

在空白水稻、小麦基质中添加低、中、高3个水平的混合标准溶液,按1.3节方法处理,每个水平平行6份,进行回收率试验,并计算相对标准偏差(RSD)。结果表明,水稻和小麦基质中真菌毒素在低、中、高3个加标水平下的平均回收率分别为88.1%~123.9%和102.0%~123.4%; RSD分别为0.2%~13.6%和0.8%~14.8%(表6)。说明方法具有良好的准确度和精密度,适用于水稻和小麦中 7种真菌毒素的定量。

**表 6:** 水稻和小麦中7种真菌毒素的加标回收率和精密度(*n*=6)

Compound	Spiked/ (μg/kg)	Rice			Wheat	
		Recovery/%	RSD/%		Recovery/%	RSD/%
AFG_1_	1.00	111.9	6.4		119.3	0.8
	2.00	110.0	8.8		110.5	3.4
	3.00	101.0	1.2		110.1	5.3
AFG_2_	1.00	113.4	1.9		117.0	7.6
	2.00	102.8	1.3		108.2	9.8
	3.00	104.5	1.2		115.9	1.6
AFB_1_	1.00	123.6	1.6		122.5	3.4
	2.00	120.6	1.9		103.7	5.2
	3.00	103.6	2.4		102.0	2.3
AFB_2_	1.00	123.5	5.9		122.0	4.5
	2.00	112.7	1.6		116.0	1.5
	3.00	100.9	8.6		109.1	3.6
OTA	4.00	123.9	10.7		120.0	9.6
	8.00	117.3	11.8		110.1	8.4
	12.0	120.2	6.9		110.4	14.8
DON	800	115.6	1.8		121.2	2.6
	1600	109.0	1.4		123.4	7.4
	2400	118.8	0.2		115.2	12.5
ZEN	40.0	105.5	9.4		120.9	7.3
	80.0	94.1	13.6		112.2	8.9
	120	88.1	12.0		107.8	10.2

### 2.7 实际样品检测

收集了浙江省46批水稻和24批小麦,按照本课题组前期建立的方法^[5]^进行筛查,并按本文的方法进行定量测定,结果46批水稻中有1批筛查出了AFB_1_和AFB_2_,筛出率为2.2%,其中含量分别为10.8 μg/kg和1.2 μg/kg, 根据本文规定的限量标准(表3),有1批超标,超标率为2.2%。24批小麦中有9批筛查出DON,筛出率为37.5%, 19批筛查出ZEN,筛出率为79.2%。根据本文规定的限量标准(表3),测定结果表明DON和ZEN均未超过限量,超标率为0。

需要进一步强调,由于MS^E^和TOF-MRM模式的侧重点不同,建议对于那些残留量低,毒性大的真菌毒素用MS^E^模式先行筛查,在筛查阳性确证后用TOF-MRM模式进行定量。以AFG_1_为例,用MS^E^模式进行筛查,按照课题组制定的筛查规则^[5]^,精确质量数的容差设为5×10^-6^,在UNIFI工作站中运行方法,结果在AFG_1_对应保留时间范围内无目标物的加合离子和碎片离子精确质量数(图5a和b),判断得出AFG_1_未筛出。

**图5:**
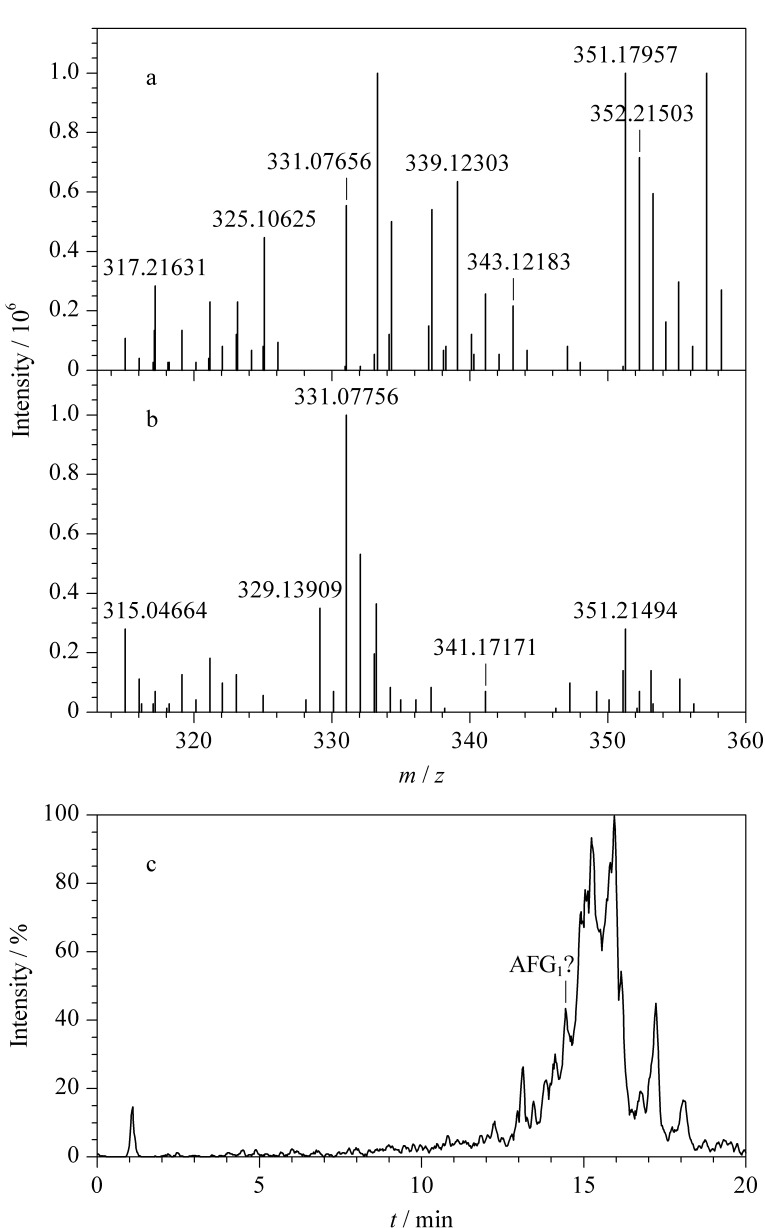
水稻样品在MS^E^模式下(a)低能量、(b)高能量质谱图 和(c)在TOF-MRM模式下的提取离子色谱图

同批样品,在TOF-MRM模式,对应保留时间处疑似有AFG_1_的色谱峰(图5c)。推测产生的原因是用TOF-MRM模式进行定量时,仪器固有条件决定了定量离子的质量数容差约为1 Da,导致基质中的一些干扰物因质量数和保留时间都与目标物接近,形成假阳性现象。

## 3 结论

本文以稻谷和小麦作为研究对象,建立了基于自建数据库的超高效液相色谱-四极杆-飞行时间质谱对水稻和小麦中7种真菌毒素定量的方法。该方法前处理操作简便,筛查精准,定量准确,适用于大批量水稻、小麦中7种真菌毒素的定量,为谷物中的真菌毒素筛查检测提供新思路与新方法,也为监管提供技术支持。
